# Molecular differences in brain regional vulnerability to aging between males and females

**DOI:** 10.3389/fnagi.2023.1153251

**Published:** 2023-05-22

**Authors:** Xianxiao Zhou, Jiqing Cao, Li Zhu, Kurt Farrell, Minghui Wang, Lei Guo, Jialiang Yang, Andrew McKenzie, John F. Crary, Dongming Cai, Zhidong Tu, Bin Zhang

**Affiliations:** ^1^Department of Genetics and Genomic Sciences, Icahn School of Medicine at Mount Sinai, New York, NY, United States; ^2^Mount Sinai Center for Transformative Disease Modeling, Icahn School of Medicine at Mount Sinai, New York, NY, United States; ^3^Icahn Institute for Data Science and Genomic Technology, Icahn School of Medicine at Mount Sinai, New York, NY, United States; ^4^Department of Neurology, Icahn School of Medicine at Mount Sinai, New York, NY, United States; ^5^Research & Development, James J. Peters VA Medical Center, Bronx, NY, United States; ^6^Neuropathology Brain Bank & Research CoRE, Icahn School of Medicine at Mount Sinai, New York, NY, United States; ^7^Alzheimer’s Disease Research Center, Icahn School of Medicine at Mount Sinai, New York, NY, United States; ^8^Ronald M. Loeb Center for Alzheimer’s Disease, Icahn School of Medicine at Mount Sinai, New York, NY, United States

**Keywords:** brain aging, gender differences, gene co-expression network, key regulators, Alzheimer’s disease

## Abstract

**Background:**

Aging-related cognitive decline is associated with brain structural changes and synaptic loss. However, the molecular mechanisms of cognitive decline during normal aging remain elusive.

**Results:**

Using the GTEx transcriptomic data from 13 brain regions, we identified aging-associated molecular alterations and cell-type compositions in males and females. We further constructed gene co-expression networks and identified aging-associated modules and key regulators shared by both sexes or specific to males or females. A few brain regions such as the hippocampus and the hypothalamus show specific vulnerability in males, while the cerebellar hemisphere and the anterior cingulate cortex regions manifest greater vulnerability in females than in males. Immune response genes are positively correlated with age, whereas those involved in neurogenesis are negatively correlated with age. Aging-associated genes identified in the hippocampus and the frontal cortex are significantly enriched for gene signatures implicated in Alzheimer’s disease (AD) pathogenesis. In the hippocampus, a male-specific co-expression module is driven by key synaptic signaling regulators including *VSNL1*, *INA*, *CHN1* and *KCNH1*; while in the cortex, a female-specific module is associated with neuron projection morphogenesis, which is driven by key regulators including *SRPK2*, *REPS2* and *FXYD1*. In the cerebellar hemisphere, a myelination-associated module shared by males and females is driven by key regulators such as *MOG*, *ENPP2*, *MYRF*, *ANLN*, *MAG* and *PLP1*, which have been implicated in the development of AD and other neurodegenerative diseases.

**Conclusions:**

This integrative network biology study systematically identifies molecular signatures and networks underlying brain regional vulnerability to aging in males and females. The findings pave the way for understanding the molecular mechanisms of gender differences in developing neurodegenerative diseases such as AD.

## Introduction

Aging-associated changes in the human brain contribute to the decline of cognitive functions and the development of various neurodegenerative disorders (Hsu et al., 2008; Lopez-Otin et al., 2013). Gender-specific changes in aging processes may contribute to the differences in the prevalence of several neurological disorders between males and females. For instance, the incidence rate of Parkinson’s disease in males is about 2-fold as in females (19.0/100K in males vs. 9.9/100K in females) (Picillo et al., 2017), while there are more females (3.3 million) living with Alzheimer’s disease than males (2.0 million) (Alzheimer’s Association, 2015; Nebel et al., 2018). Neuro-imaging studies have found many gender differences in structural changes during brain aging (Murphy et al., 1996; Raz et al., 1997; Coffey et al., 1998; Hsu et al., 2008; Kiraly et al., 2016). For example, age-associated brain volume loss in the frontal and temporal lobes is much more significant in men than women, while in the hippocampus and the parietal lobes, the loss is greater in women (Murphy et al., 1996). Another study found that the age-related decrease in brain grey matter volume at the caudate nucleus, putamen and thalamic regions is greater in men with a faster rate of decline than in women (Kiraly et al., 2016). In contrast, females have a lower white matter volume in the right deep temporal regions than males (Hsu et al., 2008). White matter alterations during aging in the precentral, cingulate, and anterior temporal regions also showed significant differences between males and females (Hsu et al., 2008). These findings suggest sex dimorphism in aging-associated microstructural changes across brain regions. However, molecular mechanisms underlying gender-specific age-related structural changes remain elusive (Flood et al., 1987; Pakkenberg et al., 2003; Bertoni-Freddari et al., 2007). The sex dimorphism at the brain morphological levels is probably induced by various molecular changes such as different hormone levels and epigenetic modifications and gene expression alterations.

There were 9 established cellular and molecular hallmarks of aging (Lopez-Otin et al., 2013), including genomic instability, epigenetic alterations, telomere attrition, mitochondrial dysfunction, proteostasis dysfunction, cellular senescence, deregulated nutrient sensing, stem cell exhaustion, and altered intercellular communication. Many studies have shown that these hallmarks are sexually dimorphic factors associated with aging (Barrett and Richardson, 2011; Dulken and Brunet, 2015; Gaignard et al., 2015; Gentilini et al., 2015; Fischer and Riddle, 2018). For example, genomic instability, such as mutation and genetic mosaicism rate, is higher in males than in females (Machiela et al., 2015; Podolskiy et al., 2016). On the other hand, aging hallmarks showed differences in gene expression patterns between males and females. Two studies of the Zebrafish brain showed that aging alters gene expression and molecular dynamics of synapses in a sexually dimorphic pattern (Arslan-Ergul and Adams, 2014; Karoglu et al., 2017). Sexually dimorphism is also found in aging-related neuroinflammation in the mouse hippocampus (Mangold et al., 2017). Compared to animal studies, very few studies have examined sex differences in human brain aging at a global molecular level. A study of the microarray data of 40 samples from 4 brain regions including entorhinal cortex (EC), hippocampus (HIPP_MA), postcentral gyrus (PCG), prefrontal cortex (PFC) and superior frontal gyrus (SFG) by Berchtold et al., showed that more genes are changed in males than in females across 4 brain regions during aging (Berchtold et al., 2008). Expression of the genes involved in energy metabolism and protein synthesis was found to decrease with aging, while immune-related genes were activated in both sexes during aging, with greater alteration in the female brains (Berchtold et al., 2008).

In this study, we aim to systematically investigate sex differences of normal aging by examining RNA-Seq data collected from 13 regions in the Genotype-Tissue Expression (GTEx) project (GTEx Consortium, 2015). We identified aging-associated gene expression patterns, gene co-expression network modules and key network drivers in male and female brains. We further studied the aging associated molecular signatures and modules shared by both genders or specific to a gender group as well as their roles in neurodegenerative disorders such as Alzheimer’s disease.

## Materials and methods

### GTEx data processing

GTEx RNA-Seq read counts and metadata were downloaded from GTEx Portal phs000424.v7.p2.^1^ Raw read counts for brain regions were extracted and a total of 1,671 samples from 13 brain regions were retained (Table 1). The demographic and neuropathological information of those subjects was summarized in Supplementary Table 1. Lowly expressed genes with expression levels of at least 1 count per million in less than 20% of samples were removed. A total of 16,494 genes were retained for further analysis. Next, normalization factors were computed on the filtered data matrix using the weighted trimmed mean of M-values (TMM) method, followed by log2 transformation and voom (Law et al., 2014) mean-variance analysis in preparation for Limma linear modeling. The normalized expression data were then split into matrices for males and females in each of the 13 brain regions.

**TABLE 1:** Summary of sample sizes for gender and age in the 13 brain regions.

Region	Region (full name)	Male			Female		
		**Young**	**Middle**	**Aged**	**Young**	**Middle**	**Aged**
AMY	Amygdala	8	29	29	5	11	18
ACC	Anterior cingulate cortex	7	36	41	6	10	21
CD	Caudate	9	52	54	8	16	21
CBH	Cerebellar hemisphere	10	45	44	5	14	18
CB	Cerebellum	13	57	50	6	17	30
CT	Cortex	10	50	48	7	18	25
FC	Frontal cortex	8	43	41	3	14	20
HIPP	Hippocampus	10	35	38	5	13	22
HTH	Hypothalamus	9	38	40	4	12	18
NAC	Nucleus accumbens	9	48	46	6	16	22
PT	Putamen	7	45	38	4	14	16
SC	Spinal cord (cervical c-1)	5	25	26	4	16	15
SN	Substantia nigra	4	26	29	5	9	15

### Identification of age-correlated genes (ACGs)

Pearson correlation coefficients were calculated between expression levels of each gene and age for males and females, respectively, in each of the 13 brain regions. *P*-values were corrected by the Benjamini-Hochberg (BH) procedure (Benjamini and Hochberg, 1995) to adjust for multiple testing. To identify the differentially expressed genes (DEGs) between aged and young brains, we split samples in each brain region into 3 groups: young adult (age < 45), middle-aged (45 ≤ age < 60) and aged adult (age ≥ 60). DEGs between each pair of the 3 groups were identified using the eBayes method to fit gene expression to a linear model implemented by the *limma* R package (Ritchie et al., 2015). Sample labels were permuted 1,000 times to obtain a null distribution of *t*-statistics across all genes. The original *t*-statistic of each gene was compared to the null distribution to calculate the FDR of that gene. Genes identified as age-positively correlated genes (APCGs) or age-negatively correlated genes (ANCGs) in at least one region for both males and females were classified as consistent APCGs (or ANCGs). Genes identified as APCGs (or ANCGs) in at least one region for either only males or only females were classified as male-specific or female-specific APCGs (or ANCGs).

To test the influence of sample size in the identification of ACGs and DEGs, we randomly chose a subset of samples from each region to identify ACGs and DEGs with the same procedure as above. From the male samples in each age group, we randomly selected the same number of samples as a corresponding female group. The samples of the 3 groups were then used to identify ACGs and DEGs with the above-described procedures. This process was repeated 1,000 times to estimate distributions for the numbers of ACG and DEG in each region. *P*-value was calculated as the number of tests with ACGs/DEGs no less than the number of ACGs/DEGs identified in the original sample set in that brain region, dividing the number of repeats (i.e., 1,000).

We also performed an interaction analysis on age and gender with all genes in addition to the analysis of each gender. We built our model as follows:


$$E\,x\,p\,r\,e\,s\,s\,i\,o\,n=\beta_{0}+\beta_{1}\,B\,M\,I+\beta_{2}\,T\,R\,I\,S\,C\,H\,D+\beta_{3}\,S\,E\,X+\beta_{4}\,A\,G\,E$$

$$+\beta_{5}\,S\,E\,X:A\,G\,E+\mu_{1}\,R\,A\,C\,E+\mu_{2}\,D\,T\,H\,H\,R\,D\,Y+\varepsilon$$

where *BMI* is Body Mass Index, *DTHHRDY* is a factor of death classification, disease or injury, leading to the cause of death listed in immediate cause of death. *TRISCHD* is the ischemic time interval between actual death, presumed death, or cross-clamp application and the start of the GTEx procedure. β indicates fixed linear effects and μ is a random effect.

### dbGaP data sets preprocessing

To validate the ACGs identified from the GTEx dataset, we downloaded an RNA-seq data set from the study of mRNA Sequencing of Human Cerebral Frontal Cortex (dbGaP Study Accession: phs001353.v1.p1) in North American Brain Expression Consortium (NABEC) (Dillman et al., 2017). Raw sequencing data were aligned to human genome HG38 using the STAR aligner (Dobin et al., 2013) and assigned to genes using the featureCounts (Liao et al., 2014). Count matrix was preprocessed and normalized using the same procedure of GTEx above. To match the age range with the GTEx cohort, individuals younger than 20 or older than 70 were excluded from the following analysis. After correcting for post-mortem interval, ACGs of the dbGaP FC were identified using the Pearson correlation for male and female, respectively. FC region male-specific APCGs and ANCGs were identified by removing APCGs and ANCGs in females, respectively, and *vice versa*.

### Human samples for validation

Formalin-fixation paraffin-embedded human brain tissues from the hippocampal formation were obtained from the Neuropathology Brain Bank & Research CoRE, in accordance with the relevant guidelines and policies of the Icahn School of Medicine at Mount Sinai (ISMMS). The experimental procedures involving human sample handling were approved by the appropriate committee at James J. Peters VA Medical Center (JJP VAMC) and ISMMS. Demographic information with 5–6 individuals/group is provided in Table 4. A comprehensive neuropathological assessment was performed on each brain to rule out the presence of a major neurodegenerative disease in excess of normal aging. Selection criteria was based on biological sex and age of death and the absence of any clinical or neuropathological neurodegenerative features. Middle-age subjects included anyone between the ages of 42–59 years old and “aged” subjects included anyone over the age of 60. Clinical exclusion criteria included dementia or movement disorder diagnosis following a comprehensive chart review. Neuropathological exclusion criteria included any macro or microscopic neurodegenerative changes (i.e., Lewy bodies, neuritic plaques, neuronal atrophy, neocortical neurofibrillary tangles, etc.) with the exception of vascular pathology and/or mild age-related changes (i.e., primary age-related tauopathy or other common age-related changes) (Crary et al., 2014).

### Real-time quantitative polymerase chain reaction (RT-qPCR)

The total RNA was extracted using the RNeasy FFPE kit following the instructions provided by the manufacture (Qiagen). The mRNA levels of genes of interest (*CD99*) were determined by RT-qPCR analysis. The *CD99* mRNAs were normalized to actin and then transformed to log2 fold changes when testing the significance of the differences between the groups using the Student’s *t*-test.

### Cell type and neuron loss rate estimation

For each sample, proportions of 6 brain cell types were inferred from the normalized gene expression data using the digital sorting algorithm (Zhong et al., 2013) with the cell markers of the 6 brain cell types extracted from the *BRETIGEA* package (McKenzie et al., 2018). Spearman correlation coefficient and *p*-value were then calculated between the proportion of each cell type and age for each brain region. The *p*-values were corrected using the BH procedure (Benjamini and Hochberg, 1995). The R package DGCA (McKenzie et al., 2016) was used to calculate the correlation difference of cell-type proportions and age between males and females. The adjusted *p*-values of the DGCA analysis were estimated using 1,000 permutations. To measure the median cell type change rate across age, we also fitted the cell type proportions with age using the non-parametric Theil–Sen estimator (Sen, 1968), which is insensitive to outliers by calculating the median of the slopes of all fitting lines through pairs of points. In addition, a down-sampling analysis was performed on the male samples for calculated the correlation coefficient 1,000 times to test whether neuron proportion was significantly correlated with age in the males with the same number of samples as the females.

### Co-expression network construction and downstream analysis

Gene co-expression networks were constructed based on normalized gene expression levels for the males or females in each of the 13 brain regions using the Multiscale Embedded Gene Co-expression Network Analysis (MEGENA) (Song and Zhang, 2015). For each gender group in each brain region, a planar-filtered network was first constructed, and then a multiscale clustering analysis was performed to identify gene co-expression modules at multiple compactness scales. Modules were then compared to random PFN modules generated by shuffling the link weights of the parent cluster to calculate statistical significance. Lastly, a multiscale hub analysis was conducted to identify highly connected hubs of each significant module. Modules with too many (>5,000) or too few (<50) genes were excluded from further analysis.

For each brain region in each gender group, genes in the identified modules were mapped with their respective rankings of correlation coefficients with age and fold changes (log2 transformed) between aged and young adults to quantify modules’ relevance with aging. The significance of a module’s association with aging was calculated using a logistic regression approach *LRpath* (Sartor et al., 2009), and *p*-values were then adjusted with Bonferroni correction. A module of a brain region in the males or females was defined as an age-associated module if it was significantly enriched with ACGs and/or aged-young DEGs in the same gender of that region.

### Gene ontology (GO) biological process and pathway enrichment analysis

GO biological process and KEGG pathway enrichment analysis was performed using the R package GO-function (Wang et al., 2012). GO biological processes with at least 1 human gene annotated in the *org.Hs.eg.db* were extracted from *GO.db* data package in *Bioconductor* version v3.5 (Huber et al., 2015). Similarly, KEGG pathways with at least one human gene annotated were extracted from the *KEGG.db* data package (Huber et al., 2015). The input for the analysis was either a set of co-expression modules or a gene signature such as age positively-correlated genes (APCGs), age negatively-correlated genes (ANCGs), up-regulated genes or down-regulated genes in aged brains versus young brains in male/female group. The *p*-value for an intersection was calculated by the hypergeometric distribution test and corrected using the BH procedure (Benjamini and Hochberg, 1995).

### Module preservation and visualization

Module preservation was calculated among modules between the male and female networks for each brain region. To identify common and unique modules, we applied the following procedures: firstly, the significance of the overlap between any two modules was calculated using FET. *P*-value was corrected by the BH procedure (Benjamini and Hochberg, 1995). We then defined set overlap as follows:


$$s\,e\,t\,o\,v\,e\,r\,l\,a\,p\,\left(A\,B\right)=\frac{2\,\left|A\,\cap B\right|}{\left|A\right|+\left|B\right|}$$

where *A* and *B* were sets of genes in two modules under consideration. Lastly, two modules were considered preserved if the adjusted FET *p*-value was less than 0.05 and the set overlap size was at least 30%. A male module and a female module were defined as preserved aging-associated modules if they were preserved and significantly enriched with ACGs and/or aged-young DEGs. Otherwise, an aging-associated module was defined as a gender-specific aging-associated module if it was not enriched with ACGs/DEGs or not preserved in the other gender network. Circos plots were employed to visualize various features of the modules in a co-expression network using the R package *NetWeaver* (Wang et al., 2016). Global co-expression networks and modules were visualized using Cytoscape (v3.3.0) (Shannon et al., 2003).

## Results

### Males are more vulnerable to brain aging with greater neuron loss and microglia gain

The sample demographics of the 13 brain regions are summarized in Table 1. The age range of the subjects is between 20 and 70. There is no significant difference in the age distribution between the two gender groups in each of the 13 brain regions (Kolmogorov–Smirnov test, *p* ≥ 0.18; Supplementary Figure 1). Notably, the number of male samples is approximately twice of female ones in each region. The neuropathological characters and confounding factors, including neurodegenerative diseases, post-mortem delay and immediate cause of death, are summarized for each brain region in Supplementary Table 1.

We first inferred the proportions of 6 brain cell types for each sample. Generally, we found that the proportion of neurons was negatively correlated with age, while the proportion of microglia was positively correlated with age (Table 2). Specifically, under an FDR cutoff of 0.05, the proportion of neurons was negatively correlated with age in the CD, CBH, CT, FC, HIPP, HTH, NAC and SC regions in the males, while in the other 5 regions, the negative correlation was non-significant with adjusted *p*-values < 0.2 (0.064–0.184) (Table 2). In contrast, the negative correlation with age was significant in 3 brain regions in the females, i.e., ACC, CT and CB. To estimate the cell type rate of changes during aging in these brain regions, we fitted the cell type proportions with age using Theil–Sen estimator. We found that neuronal loss was faster in the males than in the females in 9 brain regions, including AMY, CD, CBH, CB, HIPP, HTH, NAC, PT and SC (Supplementary Table 2), while the females showed a more neuronal loss in the CT and SN regions than the males. However, the difference was not significant in most of the regions except SC, in which males (ρ = −0.379) and females (ρ = 0.044) showed a significant difference in correlations between neuron proportions and age (adjusted *p*-value = 0.03). In contrast, the males and the females had a similar rate of increase in microglia proportion with aging across all the brain regions except CD, in which the males gained more microglia than the females. In addition, the astrocyte proportion was altered in a regional- and sex-specific manner during aging. Specifically, the astrocyte proportion increased with age in the CT region of both males and females and the male FC and CB regions. However, in the SC region, the astrocyte proportion was negatively correlated with age in the females (ρ = −0.437, adjusted *p* = 0.05) but positively correlated with age in the males (ρ = 0.253, adjusted *p* = 0.16) (Supplementary Table 2). In summary, these results indicate that brain aging is accompanied by the decreased neuron proportion and the decreased microglia proportion.

**TABLE 2:** Correlations of cell proportions and age in the males and females*.

Region	Neuron		Microglia	
	**Male ρ (adj. *p*)**	**Female ρ (adj. *p*)**	**Male ρ (adj. *p*)**	**Female ρ (adj. *p*)**
AMY	−0.324 (0.079)	−0.260 (0.091)	0.573 (0.002)	0.265 (0.051)
ACC	−0.332 (0.064)	−0.275 (0.037)	0.535 (0.003)	0.283 (0.022)
CD	−0.282 (0.012)	−0.032 (0.833)	0.296 (0.004)	0.200 (0.222)
CBH	−0.297 (0.012)	−0.089 (0.710)	−0.070 (0.530)	−0.418 (0.022)
CB	−0.185 (0.184)	−0.314 (0.003)	−0.009 (0.947)	−0.034 (0.770)
CT	−0.296 (0.012)	−0.408 (0.014)	0.087 (0.483)	0.378 (0.022)
FC	−0.236 (0.038)	−0.211 (0.342)	0.083 (0.511)	0.355 (0.051)
HIPP	−0.365 (0.038)	−0.421 (0.001)	0.562 (0.002)	0.370 (0.005)
HTH	−0.269 (0.031)	−0.222 (0.342)	0.292 (0.016)	0.548 (0.005)
NAC	−0.234 (0.038)	−0.168 (0.390)	0.197 (0.085)	0.415 (0.022)
PT	−0.158 (0.148)	−0.183 (0.390)	0.238 (0.052)	0.327 (0.085)
SC	−0.379 (0.013)	0.044 (0.833)	0.233 (0.122)	0.301 (0.102)
SN	−0.285 (0.148)	−0.218 (0.212)	0.328 (0.122)	0.018 (0.888)

*See Supplementary Table 2 in additional file 3 for the correlation of all 6 cell types.

### ACGs can be reproducibly identified in males and females

We performed a standard transcriptome-wide association study to identify genes whose expression levels were associated with age. With a false discovery rate (FDR) cutoff of 5%, correlations between gene expression and age in the 13 brain regions were calculated separately for the male and female groups (Table 3). Nine regions in the males have over 100 genes correlated with age, but only 4 regions in the females have over 100 age-correlated genes (ACGs). In the males, there were 546 to 3,709 genes whose expression levels were positively or negatively correlated with age in the AMY, CD, CBH, CB, CT, FC, HIPP and HTH regions while the numbers of ACGs in the ACC, NAC, PT and SC regions were much smaller. Notably, more than 95% of the ACGs in the SC were negatively correlated with age.

**TABLE 3:** Number of age-correlated genes identified in the males and females.

Region	Male		Female	
	**# APCGs**	**# ANCGs**	**# APCGs**	**# ANCGs**
AMY	286	387	0	0
ACC	3	2	41	61
CD	842	1442	430	530
CBH	276	270	672	683
CB	663	513	2	4
CT	534	829	468	555
FC	311	410	0	0
HIPP	1563	2146	0	1
HTH	1639	1728	0	0
NAC	1	2	0	0
PT	31	9	0	0
SC	7	187	0	0
SN	0	0	0	0

In general, there are fewer ACGs in females than in males. Specifically, we identified only 6 ACGs in the female CB region and 1 for the HIPP region. No ACG was identified in the female AMY, FC, HTH, NAC, PT, SC and SN regions. For CD and CT regions, we identified approximately 1,000 ACGs in the females, which were also less than those in males of these regions. However, we identified 102 and 1,355 ACGs in the female ACC and CBH regions, respectively, which were more than the ACG numbers in the same regions of the males though the female sample sizes were smaller. We identified a similar number of ACGs using Spearman correlation (Supplementary Table 3). These results suggested that the ACC and CBH region were probably more vulnerable in females than in males. In addition, we also identified more DEGs between the aged and young individuals in males than in females (Supplementary Table 4). To summarize, we identified more ACGs in the males than in the females across 11 brain regions except for the ACC and CBH regions.

**FIGURE 1:**
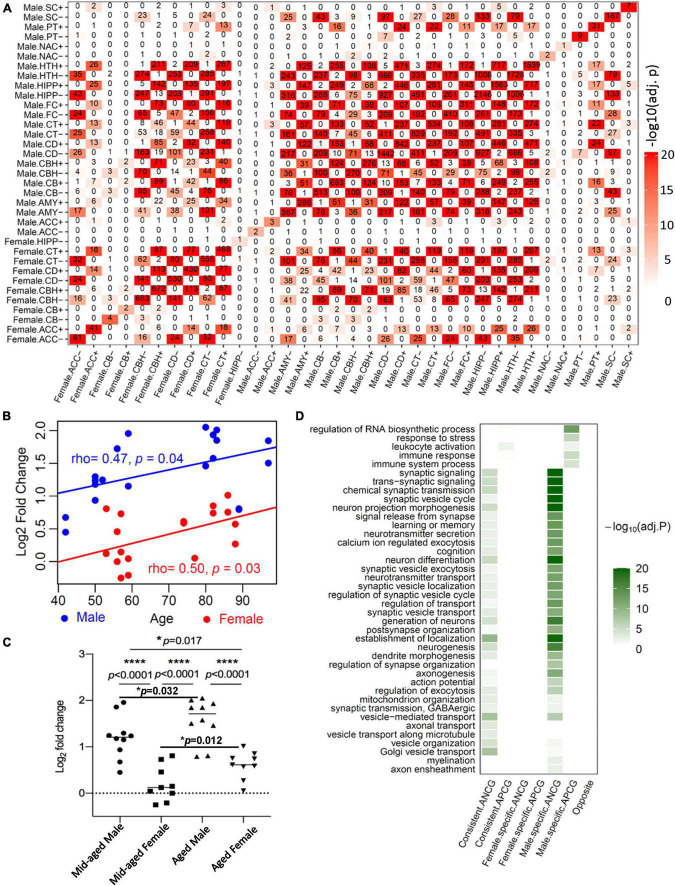
Age-correlated gene (ACG) identified in the GTEx, validation in the human hippocampus and Gene Ontology enrichment analysis of the ACGs. **(A)** The numbers of ACGs are shown in the diagonal of the heatmap. The “+” sign after the abbreviation of each brain region indicates the signatures positively correlated with age while “−” stands for those negatively correlated with age. Color intensity indicates adjusted *p*-values of the enrichment test between a pair of ACG signatures. AMY, amygdala; ACC, anterior cingulate cortex (BA24); CD, caudate (basal ganglia); CBH, cerebellar hemisphere; CB, cerebellum; CT, cortex; FC, frontal cortex; HIPP, hippocampus; HTH, hypothalamus; NAC, nucleus accumbens (basal ganglia); PT, putamen (basal ganglia); SC, spinal cord (cervical c-1); SN, substantia nigra. **(B)** The mRNA expression levels of *CD99* that were measured by RT-qPCR and normalized to the actin expression levels in each sample and log2 transformed. Pearson correlation was used to calculate the correlation coefficients between the normalized *CD99* expression levels and age in the males (blue) and females (red). **(C)** The normalized *CD99* expression levels in the middle-aged and aged healthy male and female hippocampal region. log_2_FC: 1.198 ± 0.147 in middle-aged male subjects *versus* 0.206 ± 0.127 in middle-aged female subjects; *versus* 1.584 ± 0.146 in aged male subjects; *versus* 0.606 ± 0.098 in aged female subjects; *p < 0.05, ****p < 0.0001 with Student’s *t*-test. **(D)** Top biological processes and pathways enriched with the age-positively correlated genes (APCGs) or age-negatively correlated genes (ANCGs) consistant in both males and females as well as specific to each of the 2 gender groups.

To investigate the overlap between ACGs in the males and females across the 13 brain regions, we performed Fisher’s Exact Test (FET) for ACG sets with at least one gene. Figure 1A shows the overlaps between these ACG signatures. Note that the reported significance was corrected for multiple testing. There were significant overlaps between the APCG signatures in different brain regions within each gender group and across two groups. A similar pattern was also observed for the ANCG signatures. In contrast, there was no significant overlap between the APCG and ANCG signatures across the 13 regions and two gender groups. To further compare the ACGs from the brain with ACGs signatures from other organs, we performed an enrichment analysis using Fisher’s exact test. After correcting the *p*-values using the BH procedure, we found that the APCGs and ANCGs of the brain regions were significantly enriched for their counterparts in the heart and artery (Yang et al., 2015; Supplementary Table 5). To validate the ACG signatures, we used an independent RNA-seq dataset in the FC from dbGaP (Study Accession phs001353.v2.p1). To keep the same range as the GTEx dataset, we removed individuals younger than 20 or older than 70, resulting in 102 males and 39 females for the validation study. At an FDR of 5%, 81 APCGs and 91 ANCGs were identified in the male FC, while 3 APCGs and 4 ANCGs were identified in the female FC. Among those ACGs, 23 of the 91 ANCGs and 8 of the 81 APCGs are also identified as ANGCs and APGCs in the male FC of the GTEx, which are significantly more than expected by chance (Fisher’s exact test, *p* = 3.02E-17 for the ANCGs, *p* = 1.42E-04 for the APCGs). As no ACG was identified in the female FC in GTEx, we cannot evaluate the conservation of the ACGs in the female FC. On the other hand, we also reproducibly identified 22 male-specific APCGs and 27 male-specific ANCGs in the dbGaP FC dataset, which are also significantly more than expected by chance (Fisher’s exact test, *p* = 2.54E-03 for the male-specific APCGs and *p* = 7.78E-04 for the male-specific ANCGs).

To further validate the ACGs, we collected 38 human hippocampal samples (from 11 male and 10 female subjects, subject demographic information provided in Table 4) and measured the mRNA expression levels of *CD99* using qPCR. *CD99’s* mRNA level was significantly correlated with age in both males (the CD, CT and HIPP regions) and females (the CT region) in the GTEx dataset (Supplementary Table 6A). In our validation hippocampal samples, *CD99* expression level wassignificantly correlated with age in both males (ρ = 0.47, *p* = 0.038) and females (ρ = 0.50, *p* = 0.034) (Figure 1B). Furthermore, the *CD99* expression levels were significantly higher in the aged males than the middle-aged males (*t*-test, *p* = 0.032), as well as higher in the aged females than middle-aged females (*t*-test, *p* = 0.012) (Figure 1C). In addition, *CD99* expression levels were significantly higher in males than in females, in both the middle-aged (*t*-test, *p* = 3.86E-05) and the aged group (*t*-test, *p* = 2.40E-05). In summary, the validation experiment shows that *CD99* expression level is significantly positively correlated with age in both males and females but shows a significant difference between the two sex groups, consistent with the transcriptomics based prediction. These results demonstrated the reproducibility of our ACG signatures.

**TABLE 4:** Demographic information of the subjects for validation.

Group	ID	Age	Clinical diagnosis	Neuropathological diagnosis	PMI (h)
Middle-aged female	1	56	Squamous cell carcinoma	No diagnostic abnormality	24
	2	57	Cardiomyopathy	Ischemic infarcts	24
	3	53	Severe Anemia	No diagnostic abnormality	25
	4	57	Squamous cell carcinoma	Cerebrovascular disease	24
	5	59	Acute myeloid leukemia	Cerebrovascular disease	31
Middle-aged male	6	56	Diabetes	Ischemic encephalopathy	24
	7	50	Coronary artery disease	Cerebrovascular disease	26
	8	59	Myeloproliferative neoplasms	No diagnostic abnormality	19
	9	50	Acute necrotizing pancreatitis	Cerebrovascular disease	23
	10	52	Diabetic ketoacidosis	Cerebrovascular disease	20
	11	42	End-stage renal disease	Cerebrovascular disease	58
Aged female	12	74	Adenocarcinoma	Arteriosclerosis	24
	13	86	Cardiac arrest	Primary age-related tauopathy	29
	14	82	Myocardial infarction	Primary age-related tauopathy	22
	15	77	Diabetes	Primary age-related tauopathy	14
	16	88	Metastatic adenocarcinoma	No diagnostic abnormality	7
Aged male	17	83	Adenocarcinoma	Arteriosclerosis	89
	18	80	Fibromuscular stroma	Cerebrovascular disease	16
	19	82	Cholecystitis	No diagnostic abnormality	11
	20	97	Multiple organ failure	Cerebrovascular disease	53
	21	89	Pancreatic cancer	Cerebrovascular disease	22

We then classified the ACGs across the 13 brain regions into sex-consistent ACGs, male-specific ACGs and female-specific ACGs (Supplementary Table 6A). In total, we identified 774 sex-consistent APCGs and 998 sex-consistent ANCGs. On the other hand, we identified 519 female-specific APCGs and 494 female-specific ANCGs that are correlated with age in one or more brain regions in females but not in males. In males, we identified 2,524 male-specific APCGs and 2,742 male-specific ANCGs that are correlated with age in only one or more brain regions in males but not in females. To study the functions and pathways of the ACGs, we performed a Gene Ontology enrichment analysis for each of the ACG lists. Generally, the top enriched pathways and biological processes for both sex-consistent ANCGs and male-specific ANCGs are “synaptic signaling”, “cognition”, “learning or memory”, “mitochondrion organization” and “neurogenesis” (Figure 1D and Supplementary Table 6B). Besides, the sex-consistent ANCGs are also enriched in the “axonal transport” and “vesicle transport along microtubule” biological processes, while the male-specific ANCGs are enriched in the “myelination”, “regulation of synapse organization” and “axon ensheathment” pathways (Figure 1D). On the other hand, both the male-specific APCGs and sex-consistent APCGs are enriched in the “immune response” and “immune system process” pathways. In addition, the male-specific APCGs are also enriched in the “regulation of RNA biosynthetic process” and “response to stress” pathways (Figure 1D). In contrast, females-specific ANCGs and APCGs were significantly enriched in the “cellular metabolic process” and “regulation of cell communication” pathways, respectively.

We then asked which genes were differentially correlated with age between males and females. To test whether the correlation coefficients of age and gene expression were significantly different between males and females, we performed a differential correlation analysis for each region using an R package DGCA (McKenzie et al., 2016). With a 5% FDR cutoff under 1,000 times of permutation, we identified 65 to 805 genes differentially correlated with age between males and females. We summarized the results in Supplementary Table 7.

### Identification of key gene subnetworks and regulators underlying aging in males and/or females

To gain insights into the global structures as well as the detailed local organizations of co-expression and co-regulation of the above-identified gene signatures underlying aging, we performed gene co-expression network analysis of the gene expression data from each brain region in each gender group using the multiscale embedded gene co-expression network analysis (MEGENA) (Song and Zhang, 2015). The modules, comprised of highly co-expressed genes, were first identified in each region in each gender group and were then evaluated for relevance to aging by the enrichment for respective ACG signatures.

**FIGURE 2:**
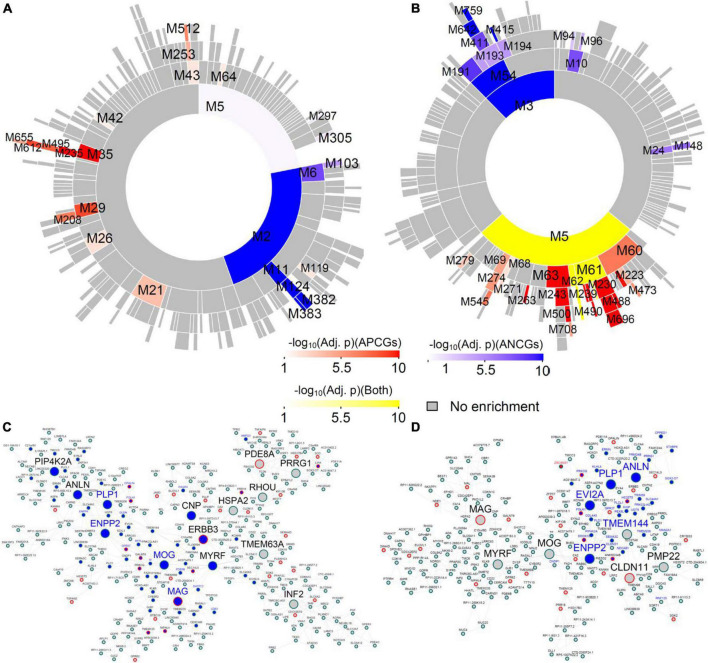
Aging-associated co-expression module shared between male and female cerebellar hemisphere (CBH). **(A)** Circos plot for the modules in the male CBH network ranked by enrichment of the ACG and DEG signatures between aged versus young males. **(B)** Circos plot for the top modules in the female CBH network ranked by enrichment of the ACG and DEG signatures in the CBH between aged versus young females. **(C)** Subnetwork of the module CBH-Male-M11 in the male CBH network, which is conserved in the female CBH network. Blue nodes are the genes whose expression levels are negatively correlated with age in the male CBH, while blue labels are the genes down-regulated in the CBH of the aged males versus that of the young males. Large nodes are the hub genes of the module. Nodes with red borders are genes up-regulated in the CBH region of the *Plp1*^–/–^ mice versus the wild-type mice. **(D)** Subnetwork of the module CBH-Female-M194 in the female CBH network. Blue nodes are the genes whose expression levels are negatively correlated with age in the female CBH, while blue labels are the genes down-regulated in the CBH of the aged females versus that of the young females. Large nodes are the hub genes of the module. Nodes with red borders are genes up-regulated in the CBH region of the *Plp1*^–/–^ mice versus the wild-type mice.

Many top-ranked, aging-associated, region-wide gene modules in the males are conserved in their respective female networks, and *vice versa* (Supplementary Tables 8–20). For instance, the module M11 of the male CBH network (encoded as CBH-Male-M11) is significantly enriched for the ANCGs in the male CBH (fold enrichment (FE) = 14.36, corrected *p* = 8.07E-53) and the down-regulated genes in aged versus young male CBH (FE = 6.48, corrected *p* = 1.69E-06) (Figure 2A). Among the 268 genes in the module CBH-Male-M11, 156 genes (66%) fall into an aging-associated female module (CBH-Female-M194) comprised of 205 genes (FE = 46.83, corrected *p* = 2.78E-252; see Supplementary Table 12). The module CBH-Female-M194 is also significantly enriched for the ANCGs in the female CBH (FE = 3.30, corrected *p* = 2.37E-31) and the down-regulated genes in aged versus young female CBH (FE = 2.63, corrected *p* = 3.86E-29) (Figure 2B). Furthermore, 6 hub genes in the module CBH-Male-M11 (Figure 2C) are also hubs of the module CBH-Female-M194 (Figure 2D), including *MOG*, *ENPP2*, *MYRF*, *ANLN*, *MAG* and *PLP1*. Both modules were significantly enriched for myelination-related biological processes (Supplementary Table 21), such as “ensheathment of neurons” and “myelination”. To further validate the shared hub genes identified in the 2 modules conserved between the males and females, we examined gene signatures in *Plp1*^–/–^ mice or *Myrf*^–/–^ mice from our previous study (McKenzie et al., 2017) and by overlaying them onto the 2 modules (CBH-Male-M11 and CBH-Female-M194). We found up-regulated genes identified from *Plp1*^–/–^ cerebellum were significantly enriched in CBH-Male-M11 (FE = 2.85, adjusted *p* = 8.77E-06, Figure 2C) and CBH-Female-M194 (FE = 3.07, adjusted *p* = 9.69E-06, Figure 2D). Similarly, down-regulated genes identified from cultured mouse *Myrf*^–/–^ oligodendrocytes were significantly enriched in CBH-Male-M11 (FE = 2.22, adjusted *p* = 1.84E-07) and CBH-Female-M194 (FE = 2.71, adjusted *p* = 8.42E-11). As myelination is an important function in the central nervous system, we further examined the myelination-associated module in the other 12 regions. We found that the myelination-associated module was significantly enriched with ANCGs and conserved across all the brain regions (Supplementary Table 22). Moreover, the hub genes in those modules are very consistent across the 13 brain regions. The most frequent hub genes in the myelination modules are *MOG*, *MYRF*, *PLP1*, *CNP* and *MAG* (Supplementary Table 22). In summary, the above results indicate that certain aging-associated processes, such as down-regulation of the myelination/nerve ensheathment modules, are well conserved between males and females across all the brain regions.

**FIGURE 3:**
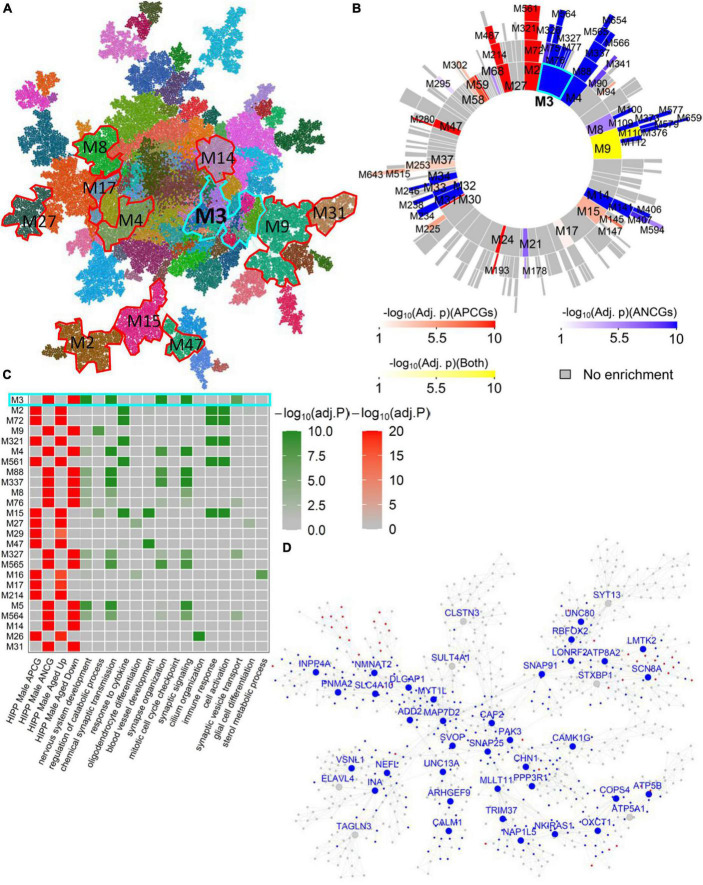
Male-specific aging-associated co-expression network in the hippocampus. **(A)** The global MEGENA network from the male hippocampus. Each color indicates a highly connected module. The top age-associated modules enriching for the ACGs and DEGs of the male hippocampus are highlighted with red and cyan frames. **(B)** Sunburst plot of the multi-scale modules in the male hippocampus network. Red blocks show the modules enriched for APCGs of the male hippocampus; blue blocks show the modules enriched for ANCGs of the male hippocampus; yellow blocks show the modules enriched for both APCGs and ANCGs. **(C)** Heatmap of the top 25 modules in the male hippocampus network enriched with ACGs. The left panel shows the adjusted *p*-values of ACG and DEG enrichments of the 25 modules. The right panel shows the adjusted *p*-values of enrichment for the top 2 Gene Ontology biological processes in each of the 25 modules. **(D)** Subnetwork of the top age-associated module Hippocampus-Male-M3. Nodes with blue labels are ANCG hub genes in the male hippocampus. Red and blue nodes are up- and down-regulated genes in aged males versus young males, respectively.

In the AMY, HIPP, HTH and FC brain regions, we observed many male aging-associated modules that don’t overlap with female aging-associated modules (Supplementary Tables 8–20). For instance, in the male hippocampal network (Figure 3A), Hipp-Male-M3 (Figures 3B, C) was significantly enriched for the ANCGs (FE = 3.22, corrected *p* = 5.71E-174) and the down-regulated DEGs between aged versus young hippocampus (FE = 3.63, corrected *p* = 3.50E-142). These genes in the module Hippocampus-Male-M3 were implicated in the pathways such as “nervous system development”, “synaptic signaling” and “neuron development” (Supplementary Table 21). Moreover, top hub genes in this module (Figure 3D) were associated with the development of Alzheimer’s disease, including *VSNL1* (Kirkwood et al., 2016), *INA* (Dickson et al., 2005), *CHN1* (Kato et al., 2015), *NMNAT2* (Ljungberg et al., 2012), and *MAP7D2* (Khundakar et al., 2016). In particular, *INA*, *VSNL1*, *MYT1L* and *MAP7D2* were identified as male-specific aging-associated hub genes across multiple brain regions (see Supplementary Tables 6A, 8–20). In addition to the Hipp-Male-M3, we also identified many modules specific to the male gene coexpression networks (Supplementary Tables 8–20), such as the modules HIPP-Male-M9, CB-Male-M48, SC-Male-M100, and HIPP-Male-M27. These modules are significantly enriched for the glia and neuron transmission functions such as “regulation of catabolic process”, “glial cell differentiation”, “vesicle-mediated transport in synapse”, and “chemical synaptic transmission” (Supplementary Table 21).

**FIGURE 4:**
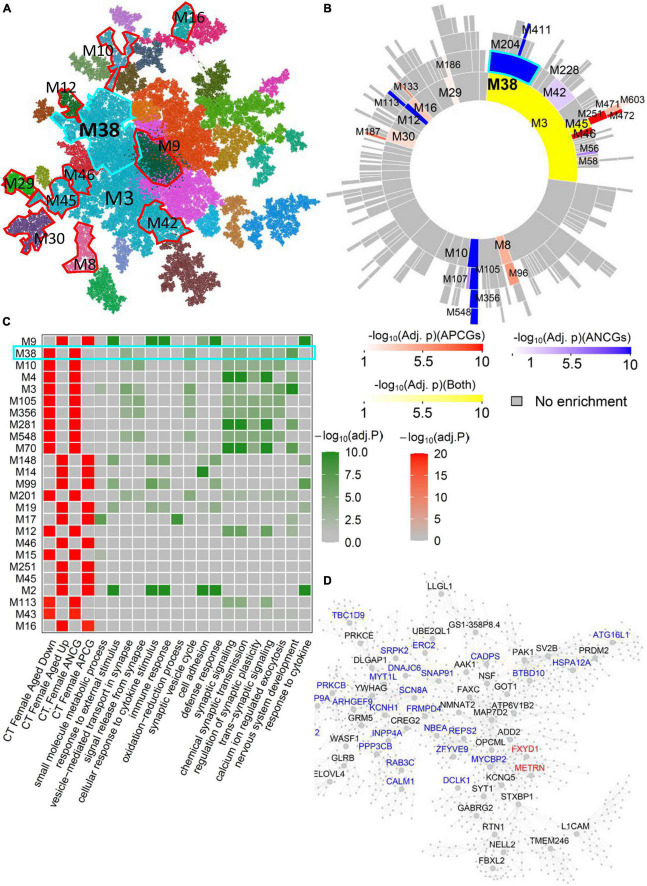
Female-specific aging-associated co-expression network and modules in the cortex. **(A)** The global MEGENA network from the female cortex. Each color indicates a highly connected module. The top age-associated modules enriching for the ACGs and DEGs of the female cortex are highlighted with red and cyan frames. **(B)** Sunburst plot of the multi-scale modules in the female cortex network. Red blocks show the modules enriched for APCGs of the female cortex; blue blocks show the modules enriched for ANCGs of the female cortex; yellow blocks show the modules enriched for both APCGs and ANCGs. **(C)** Heatmap of the top 25 modules in the female cortex network enriched with ACGs. The left panel shows the adjusted *p*-values of ACG and DEG enrichments of the 25 modules. The right panel shows the adjusted *p*-values of enrichment for the top 2 Gene Ontology biological processes in each of the 25 modules. **(D)** Subnetwork of the module CT-Female-M38. Red and blue nodes represent APCG and ANCG hub genes in the female cortex, respectively.

Similarly, female-specific aging-associated modules have been identified in other brain regions such as CD and CT. For example, several modules in the female CT network (Figure 4A) were significantly enriched for the ACG and DEG signatures in the female CT without significant overlap with the aging-associated module in the male CT network (Supplementary Table 13). For example, CT-Female-M38, the 2nd module most associated with aging (Figures 4B, C), was significantly enriched for the ANCGs in the female CT (corrected *p* = 1.75E-63) and the down-regulated DEGs in CT between aged versus young females (corrected *p* = 3.89E-72) (Figure 4D). The genes in the module CT-Female-M38 were associated with “neuron projection morphogenesis” (FE = 2.25, corrected *p* = 3.67E-06) and “axon development” (FE = 2.31, corrected *p* = 6.91E-05) (Supplementary Table 21), and regulated by female-specific key drivers include *SRPK2*, *REPS2* and *FXYD1*. Moreover, there are also many female-specific modules such as CT-Female-M10, NAc-Female-M6, AMY-Female-M25 and CT-Female-M148 (Supplementary Tables 8–20) and they are enriched for pathways like calcium ion regulated exocytosis, protein targeting to ER, inflammatory response, and cellular response to cytokine stimulus (Supplementary Table 21).

### Aging-related modules and key genes are associated with Alzheimer’s diseases

**FIGURE 5:**
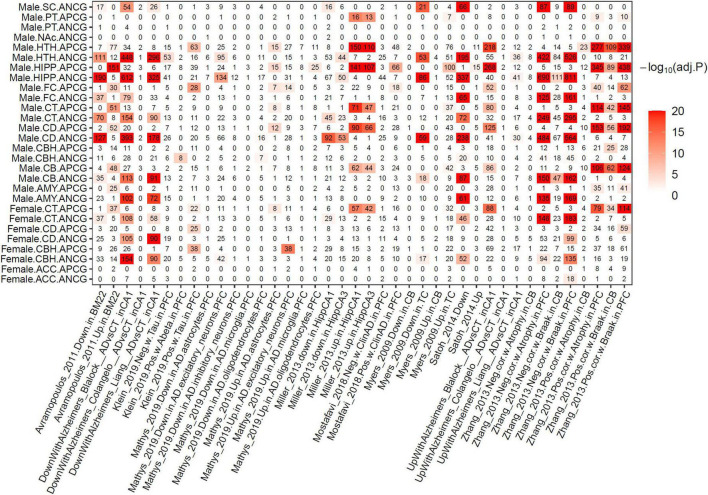
Overlapping between ACGs and Alzheimer’s disease gene signatures of previous studies. The numbers in the heat map show the shared gene numbers. Color intensity indicates the adjusted *p*-value of Fisher’s Exact Test.

To investigate the association between normal brain aging and Alzheimer’s disease (AD), we performed an enrichment analysis between the ACG signatures and previous AD gene signatures (Colangelo et al., 2002; Liang et al., 2008; Webster et al., 2009; Avramopoulos et al., 2011; Blalock et al., 2011; Szymanski et al., 2011; Miller et al., 2013; Zhang et al., 2013; Satoh et al., 2014; Mostafavi et al., 2018; Klein et al., 2019; Mathys et al., 2019). As shown in Figure 5, the ACG signatures identified from various brain regions were significantly enriched for the AD signatures identified from previous studies. More importantly, the APCG signatures were significantly enriched for the genes positively correlated with AD phenotypes (Braak staging and atrophy) or genes up-regulated in AD versus control, while the ANCG signatures were significantly enriched for the genes negatively correlated with AD phenotypes or down-regulated in AD. Specifically, the APCGs in the male FC, CB, CBH, CD, CT, HIPP, HTH and AMY regions were significantly enriched for the genes positively correlated with Braak stages and brain atrophy in PFC and CB identified by Zhang et al. (2013) and the genes up-regulated with AD versus control in the HIPP CA1 and CA3 sub-regions. On the other hand, the ANCGs in the male AMY, CB, CD, CT, FC, HIPP and HTH regions significantly overlapped the genes negatively correlated with Braak stages and brain atrophy in the PFC (Zhang et al., 2013) and genes down-regulated in thalamocortical radiations, superior temporal gyrus and hippocampal CA1 and CA3 regions of AD brains (Webster et al., 2009; Szymanski et al., 2011; Miller et al., 2013). In summary, AD and aging showed many consistent transcriptomic alterations as aging is the key vulnerability for AD development.

Interestingly, 134, 95 and 42 down-regulated genes identified from AD PFC excitatory neurons in the Mathys et al. single-cell study (Mathys et al., 2019) were significantly enriched for the ANCGs in the male HIPP (corrected *p* = 9.44E-11), male HTH (corrected *p* = 1.57E-4) and female CBH (corrected *p* = 0.015) regions, respectively. On the other hand, 38 and 14 up-regulated genes in AD excitatory neurons were enriched for the APCGs in the male FC (corrected *p* = 2.18E-14) and the female CBH (corrected *p* = 1.31E-03) regions, respectively. By contrast, dysregulated genes in AD inhibitory neurons showed no enrichment for the ACGs. Furthermore, the up-regulated genes in AD astrocytes were significantly enriched for the APCGs in six brain regions, including CB, CD, CT, FC, HIPP and HTH. The results suggested that aging may have stronger effects on excitatory neurons and astrocytes than other cell types during AD development. Thus, aging effects on excitatory neurons and astrocytes may increase vulnerability to AD development and progression.

## Discussion

Previous studies showed that the proportion of neuronal loss varies not only in different brain regions (ranging from no more than 10% (Pannese, 2011) to 50% (Devaney and Johnson, 1980), but also in different neuron subtypes (Hua et al., 2008). Our data shows that ACGs are enriched for the genes differentially expressed in AD excitatory neurons in comparison with control, but not those differentially expressed in inhibitory neurons, confirming that the loss of neurons in certain neuron subtypes is more severe than others during aging. Morphological studies showed that synaptic function was also significantly altered during aging (Pannese, 2011), with a decrease in dendrites and axons as well as the loss of dendritic spines and myelin sheaths. Moreover, transcriptomic analyses indicate that synapse-related genes and co-expression modules are extensively down-regulated across different brain regions (Berchtold et al., 2013; Dillman et al., 2017). In this study, we showed that the males have faster neuronal loss rates during aging than the females in 9 brain regions (including AMY, CD, CBH, CB, HIPP, HTH, NAC, PT and SC), while the females had a faster rate of neuronal loss in the CT and SN regions during aging. This supports the previous findings that males are generally aging faster than the females measured by many aging hallmarks (Barrett and Richardson, 2011; Dulken and Brunet, 2015; Gaignard et al., 2015; Gentilini et al., 2015; Fischer and Riddle, 2018). Further studies should be designed to investigate the gender differences in proteostasis dysfunction, cellular senescence, deregulated nutrient sensing and altered intercellular communication during aging since no study focuses on the gender differences of these 4 hallmarks. Opposite to the decreased neuron proportion during aging, the microglia proportion increases with age in most of the brain regions in both gender groups. The role of increased microglia proportion in the brain during aging and its contribution to gender differences in brain aging await further investigation.

In this study, we identified more aging-associated genes in the male HIPP, HTH, FC, CD, AMY and CB brain regions than in the respective female regions. This is consistent with a prior study, which showed that subcortical regions in males were aging faster than in females (Kiraly et al., 2016). In contrast, we identified more ACGs in the female ACC and CBH brain regions than the respective male ones, suggesting that sex differences in aging-associated gene expression changes are region-specific. In the CT, CD and CBH regions, hundreds of ACGs were identified in both males and females, while both gender groups have only a few or no ACGs in the NAC, PT, SC and SN regions. As there are twice as many males than females in most of the brain regions in the current GTEx data, the statistical power is larger in the male group, which may contribute to more ACGs identified in the male brain regions than the corresponding female ones. Nevertheless, the CBH region in the females has more ACGs than the males, suggesting that the CBH may age faster in the females than the males, which is further supported by the finding of the increased proportion of microglia in the females than the males. Using an independent human dataset from the dbGap and our hippocampal RT-qPCR cohort, we reproducibly identified many ACGs in the GTEx and validated CD99 as a ACG in both male and female. Due to the relatively low statistical power in the female group in the GTEx cohort, many ACGs in the females are yet to be identified.

Age-related gene expression changes across the central nervous system may contribute to the development of neurodegenerative disorders and functional deficits. Understanding the normal brain aging process helps elucidate the contribution of aging to neurodegenerative disorders and impairment of the brain and offers the potential to prevent, mitigate, and even reverse the impairment with potential therapeutics targeting the dysregulated pathways (Ali et al., 2017). Previous studies suggested that age-related cognitive decline was associated with mTOR signaling, chromatin modification, oxidative stress and dysregulation of mitochondrial function (Bishop et al., 2010; Wyss-Coray, 2016; Mostafavi et al., 2018). These changes during aging could account for the vulnerability of neurons to neurodegenerative stressors because of their high energetic demands (Bishop et al., 2010). Indeed, many key drivers in the aging-related modules identified in both sexes have been demonstrated to contribute to the development of AD and other neurodegenerative disorders. For instance, our study shows that a gene encoding nicotinamide nucleotide adenylyltransferase 2 (*NMNAT2*), a critical enzyme in the NAD biosynthetic process, is significantly down-regulated during aging in both the males and females. This finding is consistent with the decreased expression of *NMNAT2* in Alzheimer’s, Huntington’s, and Parkinson’s diseases (Ali et al., 2016, 2017). The decreased expression of *NMNAT2* would reduce the biosynthesis of NAD and then decrease bioenergy generation, which may contribute to the vulnerability of neurons. However, we still do not know the roles of many key drivers identified in this study in the development of neurodegenerative diseases, such as *REPS2* and *FXYD1*.

Estrogen acts as anti-aging-hallmark roles in the brain, such as promoting mitochondrial function, elevating DNA repair enzymes and increasing synaptic plasticity (Zarate et al., 2017). In the GTEx cohort, the genes coding estrogen receptors (*ESR1* and *ESR2*) and aromatase (*CYP19A1*, *HSD17B1* and *HSD17B2*) were lowly expressed in the 13 brain regions studied here and they were not correlated with age. For their low expression levels, *ESR2, HSD17B2* and *CYP19A1* were excluded from the further analyses. This suggested that brain neurons are probably affected by the estrogen level decreasing in blood, especially in females after menopause. This should be confirmed by providing more pieces of evidence in future brain aging studies.

Nevertheless, this study has some limitations. Firstly, estimation of the cell proportions from the bulk tissue RNA-seq data was based on the expression levels of the known marker genes of six brain cell types. The age-associated changes of cell type proportions in males and females need be further investigated with single-cell RNA-Sequencing data with sufficient number of young, middle-aged and aged brains. Secondly, the differences in epigenetic alterations (Nativio et al., 2018; Klein et al., 2019) between males and females are important sources of the gender differences in gene expression changes during aging. What are the gender-specific epigenetic alterations during brain aging? What are the roles of those gender-specific aging epigenetic alterations in neurodegenerative disease? These questions need more data to answer in future studies of gender differences in brain aging. Next, although we did not find a significant interaction between age and gender due to the relatively sample size there may be interactions between age and gender if enough samples are obtained. Lastly, as there were twice as many males as females in most of the brain regions in the current GTEx data, the statistical power is larger in the male group, which may contribute to higher numbers of ACGs identified in the male brain regions. Nevertheless, the CBH region in the females showed more ACGs than the males, suggesting that the CBH may age faster in the females than the males, which is further supported by the finding of the increased proportion of microglia in the females than the males.

## Conclusions

Dramatic differences in brain cell type proportion and gene expression changes during aging between males and females were observed in several brain regions. Key molecular networks and targets underlying regional vulnerability to aging in males and females were further identified. These findings pave the way for understanding the molecular mechanisms of gender differences in aging and developing neurodegenerative diseases such as AD.

## Data availability statement

The original contributions presented in this study are included in the article/Supplementary material, further inquiries can be directed to the corresponding author.

## Ethics statement

The studies involving human participants were reviewed and approved by Ethics Committee at James J. Peters VA Medical Center (JJP VAMC) and Icahn School of Medicine at Mount Sinai. The patients/participants provided their written informed consent to participate in this study.

## Author contributions

BZ conceived and designed the project. XZ, MW, LG, AM, and JY collected the data. XZ analyzed the data. JC, LZ, KF, DC, and JFC performed RT-qPCR validation. XZ, JC, LZ, MW, LG, ZT, DC, and BZ wrote and edited the manuscript. All authors read and approved the final manuscript.

## Footnotes

1
https://www.gtexportal.org/home/datasets
