# Supplementary material for: Molecular differences in brain regional vulnerability to aging between males and females
Source: Front Aging Neurosci. 2023 May 22;15:1153251. doi: 10.3389/fnagi.2023.1153251 (PMC10239962; doi:10.3389/fnagi.2023.1153251)
Supplement: Supplementary file 1 [file Table_1.DOCX]

# Supplementary Material

**Additional file 1. Supplementary Results**. **Figure S1.** Age distributions of the male and female subjects in the GTEx brain dataset. **Figure S2.** Heatmap of overlap among the DEGs identified from comparison of aged and young adults in males and females. **Table S3.** Number of ACGs identified by Spearman correlation analysis. **Table S4.** Number of DEGs between aged and young individuals in the males and females. **Table S22.** Myelination modules in the males and females across 13 brain regions. **Table S23.** Statistics of ACGs by down-sampling male samples. **Table S24.** Statistics of DEGs in subsets of male samples in aged versus young subjects. **Table S25.** Number DEGs in middle aged versus young individuals. **Table S26.** Number DEGs in aged versus middle aged individuals. (DOCX)

**Additional file 2. Table S1.** Summary of neuropathological characters and confounding factors of the male and female donors. (XLSX)

**Additional file 3. Table S2.** Correlation between proportion of 6 brain cell types and age in the males and females. (XLSX)

**Additional file 4. Table S5.** Enrichment of ACGs in brains and ACGs of other tissues. (XLSX)

**Additional file 5. Table S6.** Age correlated genes across the 13 brain regions in the males and females. **(**XLSX**)**

**Additional file 6. Table S7.** Genes differentially correlated with age in the males and females identified using the DGCA package. (XLSX)

**Additional file 7. Table S8-S20.** Region-wide modules conserved between males and females or specific to a gender group. (XLSX)

**Additional file 8. Table S21.** Most significantly enriched Gene Ontology Biological Processes of the top ranked 25 aging-associated modules in each brain region (XLSX)
