# Supplementary material for: Molecular differences in brain regional vulnerability to aging between males and females
Source: Front Aging Neurosci. 2023 May 22;15:1153251. doi: 10.3389/fnagi.2023.1153251 (PMC10239962; doi:10.3389/fnagi.2023.1153251)
Supplement: Supplementary file 2 [file Table_2.DOCX]

**Table of Contents**

**Supplementary Results:**

**Down-sampling in males did not significantly reduce the age-correlated genes (ACG) numbers in most of the brain regions**

**ACGs identified by Spearman correlation**

**MEGENA network analysis by excluding X- and Y-chromosome genes**

**Males showed more differentially expressed genes (DEGs) than females in aged versus young individuals**

**Neuron proportion and age correlation did not significantly change after down-sampling males**

**Supplementary Figure:**

**Figure S1. Age distributions of the male and female subjects in the GTEx brain dataset.**

**Figure S2. Heatmap of overlap among the DEGs identified from comparison of aged and young adults in males and females**

**Supplementary Tables:**

**Table S3. Number of ACGs identified by Spearman correlation analysis**

**Table S4. Number of DEGs between aged and young individuals in the males and females**

**Table S24. Myelination modules in the males and females across 13 brain regions**

**Table S25. Statistics of ACGs by down-sampling male samples**

**Table S26. Statistics of DEGs in subsets of male samples in aged versus young subjects**

**Table S27. Number DEGs in middle aged versus young individuals**

**Table S28. Number DEGs in aged versus middle aged individuals**

**Down-sampling in males did not significantly reduce the ACG numbers in most of the brain regions**

The disparity in ACG sizes between male and female could be partly if not all attributed to the sample size difference. To test whether the ACGs in males can be identified with a smaller sample size as in females, we down-sampled the male samples to the same samples size as female samples in each brain regions and identified ACGs with the same approach. Indeed, we identified a smaller number of ACGs on average using smaller sample sizes (**Supplementary Table S23**). Specifically, in the brain regions CBH, FC, HIPP and HTH, the number of ACGs was significantly reduced with down-sampling (*p* < 0.05). In contrast, there was not significant difference in average ACG number with down-sampling comparing to the original number. To summarize, a slightly decreased number of ACGs were identified by down-sampling but the number of ACGs were still larger in the males than those in the females.

**ACGs identified by Spearman correlation**

We further identified ACGs in the males and females using Spearman correlation which can capture the non-linear relationship and better handle outliers. Comparing to Pearson correlation analysis, more ACGs were identified in the ACC, CBH, CB, CT, FC, HIPP, NAC, SC regions in the males (**Supplementary Table S4**), while less ACGs were identified in the AMY and HTH regions. By contrast, there were significantly less ACGs identified in the ACC, CD and CBH regions using Spearman correlation analysis. But in the CT and HIPP regions, more Spearman based ACGs were identified than the Pearson based ones. In summary, Spearman and Pearson correlations complement each other.

**MEGENA network analysis by excluding X- and Y-chromosome genes**

To test whether the sex-specific age-associated modules are a result of ACGs on sex chromosomes, we excluded the X- and Y- chromosome genes when analyze the aging-associated modules. Without the sex chromosome genes, the rank of the age-associated modules was slightly different from the original rank and the significance levels of the age-associated modules decrease slightly too. Even so, we still identified similar sex-specific age-associated modules. For example, the female-specific age-associated module CT-Female-M38 was significantly enriched for the female ANCGs (adjusted *p* = 1.75E-63) and ranked at the 2^nd^. After excluding the genes on the sex chromosomes, the module CT-Female-M38 is still ranked as the 2^nd^ but the significance level decreased slightly (adjusted *p* = 4.03E-58). In summary, excluding the sex chromosome genes has some small impact on the significance and ranking of the age-associated modules, but it does not change the overall conclusions.

**Males showed more DEGs than females in aged versus young individuals**

To further investigate brain aging- associated gene expression changes in the males and females, we focused on identifying differentially expressed genes (DEGs) between aged (age ≥ 60) and young (age < 45) subjects in each gender group for each brain region. In the brain regions AMY, CD, CB, CT, FC, HIPP and HTH, there were more DEGs identified between old and young subjects in the males than those of the females (**Supplementary Table S6**). CBH was the only region with more DEGs identified in females when compared to DEGs of males. There are significant overlaps in up-regulated genes of aged versus young subjects in different brain regions (**Supplementary Figure S2**). Such overlaps can be seen when comparing males and females. These results indicate some commonly shared molecular signature changes during aging in different brain regions and between the males versus females.

We also performed differential expression analysis with down-sampling from corresponding groups for each brain region. With the same cutoff, we identified smaller number of DEGs between brain regions of aged and young adults on average in 1,000 repeats (**Supplementary Table S24**). In HIPP and HTH regions, we identified significantly fewer DEGs in the down-sampling comparing to the original number of DEGs. On the other hand, we identified more DEGs in brain regions ACC, FC, NAC, PT, SC and SN comparing to the original numbers as there is no DEGs identified in the original whole data set. No significant difference was found in other 5 brain regions.

When comparing the gene expression patterns between middle-aged and young subjects, we identified 598 up- and 373 down-regulated genes in male CD, respectively, while in AMY, CB and HTH regions we only identified a few DEGs (**Supplementary Table S25**). In contrast, in the females, we identified 1,196 up- and 1,141 down-regulated genes in CBH, respectively, with only a few DEGs identified in CD and no DEGs identified in other 11 brain regions. For the comparison between aged and middle aged subjects, over 1,000 DEGs were identified in HIPP and NAC regions of the males (**Supplementary Table S26**) while only a few DEGs were identified in the CD, CBH, CT and HTH regions. However, in the females, no DEG was identified between aged and middle aged subjects, except 3 DEGs identified in CT region (**Supplementary Table S26**).

**Neuron proportion and age correlation did not significantly change after down-sampling males**

The correlation between neuron proportion and age in males is not due to a larger sample size compared to female samples. When we reduced the sample size, the correlation remained significant in the 11 regions of males in 211 to 874 of 1,000 repeats.


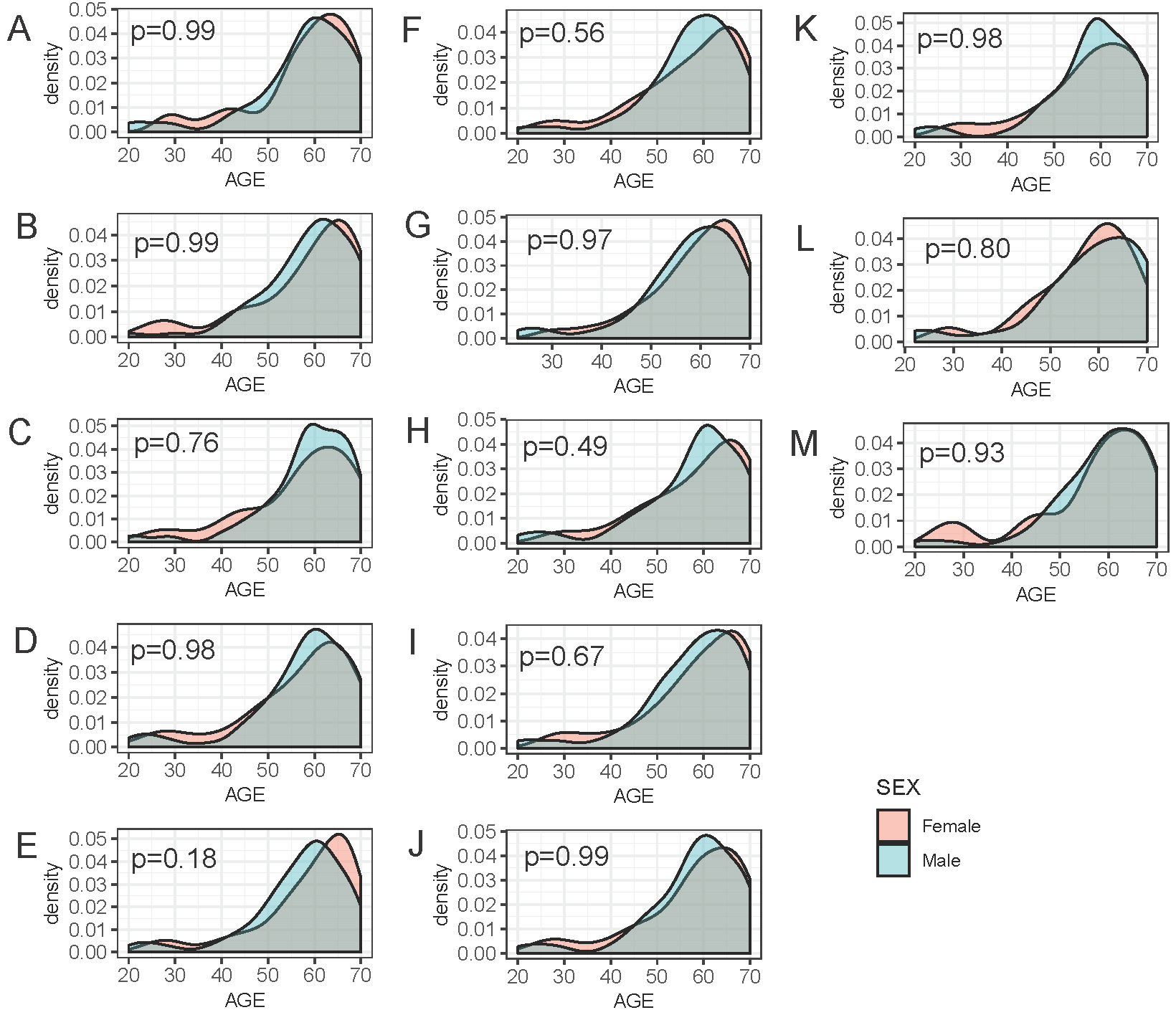


**Figure S1. Age distributions of the male and female subjects in the GTEx brain dataset.** Age distributions for the male and female samples in brain regions (**A**) Amygdala; (**B**) Anterior cingulate cortex (BA24); (**C**) Caudate (basal ganglia); (**D**) Cerebellar Hemisphere; (**E**) Cerebellum; (**F**) Cortex; (**G**) Frontal Cortex; (**H**) Hippocampus; (**I**) Hypothalamus; (**J**) Nucleus accumbens (basal ganglia); (**K**) Putamen (basal ganglia); (**L**) Spinal cord (cervical c-1); (**M**) Substantia nigra, . The *p*-values between the males and females are calculated using the Kolmogorov–Smirnov test.


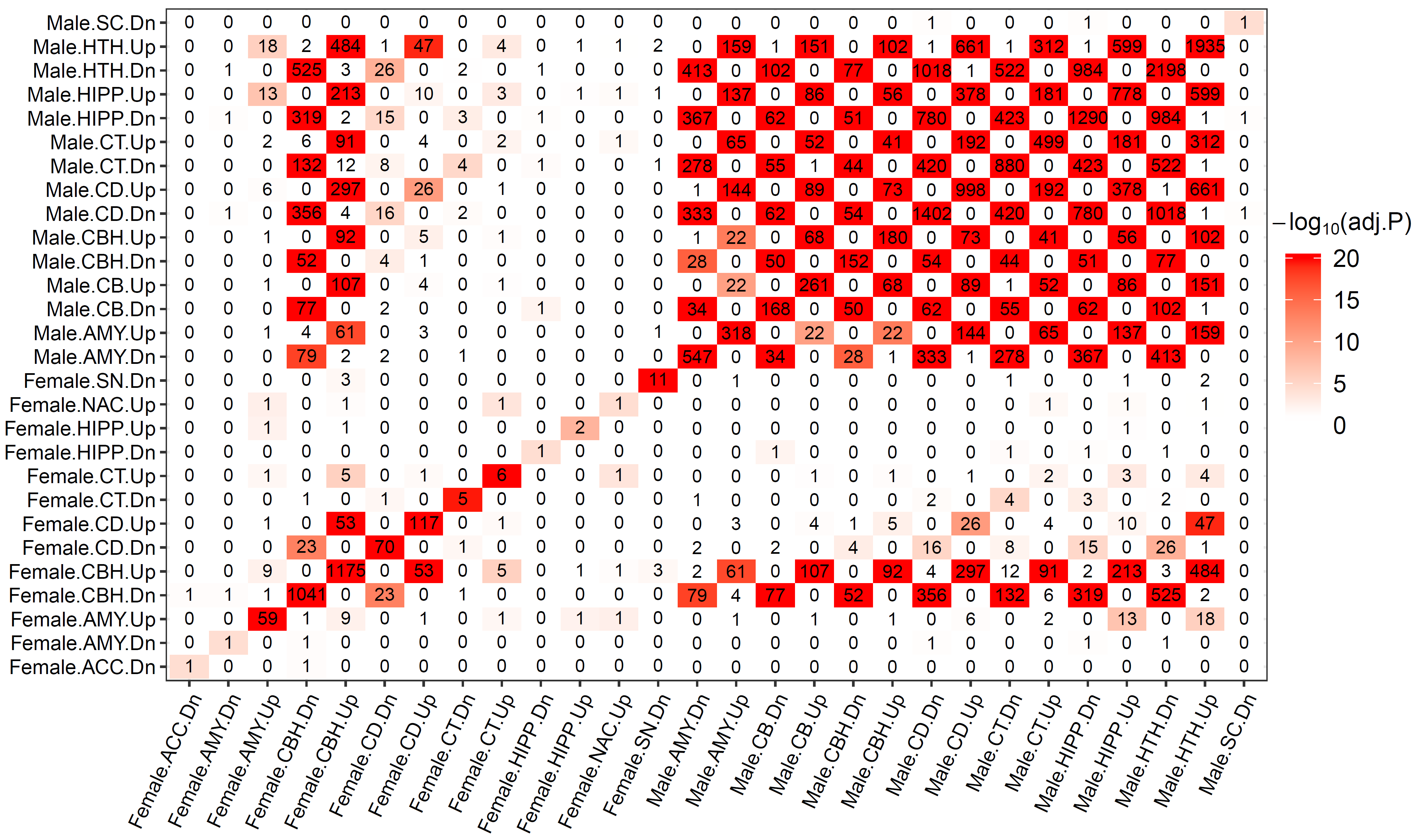


**Figure S2. Heatmap of overlap among the DEGs identified from comparison of aged and young adults in males and females.** The numbers of DEGs are shown in diagonal of the heat map. In the names of the DEG signatures, “Up” indicates up-regulation in the aged group versus the young one while “Dn” indicates down-regulation in the aged versus young adult brains. Color intensity indicates adjusted *p*-values of the enrichment test for any two DEG sets. AMY, Amygdala; ACC, Anterior cingulate cortex (BA24); CD, Caudate (basal ganglia); CBH, Cerebellar Hemisphere; CB, Cerebellum; CT, Cortex; FC, Frontal Cortex; HIPP, Hippocampus; HTH, Hypothalamus; NAC, Nucleus accumbens (basal ganglia); PT, Putamen (basal ganglia); SC, Spinal cord (cervical c-1); SN, Substantia nigra.

**Supplementary Tables**

**Table S3. Number of ACGs identified by Spearman correlation analysis**

| **Brain Region** | Male | | Female | |
| --- | --- | --- | --- | --- |
|  | APCGs | ANCGs | APCGs | ANCGs |
| Amygdala | 4 | 9 | 0 | 0 |
| Anterior_cingulate_cortex_BA24 | 39 | 13 | 8 | 5 |
| Caudate_basal_ganglia | 918 | 1,160 | 0 | 0 |
| Cerebellar_Hemisphere | 915 | 648 | 113 | 156 |
| Cerebellum | 972 | 806 | 8 | 18 |
| Cortex | 616 | 1,049 | 1,147 | 1,477 |
| Frontal_Cortex | 463 | 565 | 0 | 0 |
| Hippocampus | 2,534 | 2,952 | 37 | 22 |
| Hypothalamus | 1,611 | 1,517 | 2 | 0 |
| Nucleus_accumbens_basal_ganglia | 22 | 25 | 7 | 3 |
| Putamen_basal_ganglia | 50 | 25 | 0 | 0 |
| Spinal_cord_cervical_c-1 | 0 | 0 | 0 | 0 |
| Substantia_nigra | 0 | 0 | 0 | 0 |

**Table S4. Number of DEGs between aged and young subjects in the males and females**

| **Region** | **Male** | | **Female** | |
| --- | --- | --- | --- | --- |
|  | **# Up DEGs** | **# Down DEGs** | **# Up DEGs** | **# Down DEGs** |
| AMY | 318 | 547 | 59 | 1 |
| ACC | 0 | 0 | 1 | 1 |
| CD | 998 | 1402 | 117 | 70 |
| CBH | 180 | 152 | 1175 | 1041 |
| CB | 261 | 168 | 0 | 0 |
| CT | 499 | 880 | 6 | 5 |
| FC | 0 | 0 | 0 | 0 |
| HIPP | 778 | 1290 | 2 | 1 |
| HTH | 1935 | 2198 | 0 | 0 |
| NAC | 0 | 0 | 1 | 0 |
| PT | 0 | 0 | 0 | 0 |
| SC | 0 | 1 | 0 | 0 |
| SN | 0 | 0 | 11 | 0 |

**Table S22. Myelination modules in the males and females across 13 brain regions**

| Male module | adj.p * | Male hub genes | Female module | adj.p * | Female hub genes | Overlap |
| --- | --- | --- | --- | --- | --- | --- |
| AMY-M8 | 1.44E-104 | MOG, MYRF, PLP1, CNP | AMY-M322 | 1.93E-46 | MOG, MYRF, PLP1, CNP, MAG | 0.424 |
| ACC-M4 | 9.92E-07 | MOG, MYRF, PLP1 | ACC-M29 | 5.74E-19 | MOG, MYRF | 0.376 |
| CD-M11 | 9.45E-04 | MOG, CNP, PLP1, MYRF | CD-M9 | 1.17E-04 | MOG, CNP, PLP1, ABCA2 | 0.430 |
| CBH-M341 | 6.02E-13 | MOG, CNP, MYRF, MAG | CBH-M5 | 1.37E-126 | MOG, CNP, MYRF, MAG | 0.600 |
| CB-M11 | 2.76E-87 | MOG, MYRF, PLP1, MAG | CB-M194 | 2.37E-31 | MOG, MYRF, PLP1, MAG | 0.660 |
| CT-M4 | 4.58E72 | MOG, MYRF, CNP, MAG | CT-M80 | 0.14 | MOG, MYRF, MAG | 0.316 |
| FC-M18 | 1.61E-19 | MOG, MYRF, CNP, MAG | FC-M8 | 1.00E-05 | MOG, MYRF, CNP, MAG | 0.515 |
| HIPP-M151 | 1.84E-27 | MOG, CNP, MYRF | HIPP-M135 | 1.00E-05 | MOG, CNP, GJC2 | 0.302 |
| HTH-M3 | 0.09 | MOG, MYRF, CNP, MAG | HTH-M5 | 0.40 | MOG, MYRF, CNP, MAG | 0.507 |
| NAC-M3 | 0.58 | MOG, MYRF, CNP, MAG | NAC-M5 | 2.31E-50 | MOG, MYRF, CNP, MAG | 0.385 |
| PT-M2 | 6.28E-125 | MOG, MYRF, CNP, MAG | PT-M7 | 5.06E-04 | CENPB, SEPT7, ZNF664, ACBD5 | 0.303 |
| SC-M14 | 1.46E-10 | MYRF, MAG, GABPB2 | SC-M5 | 2.09E-228 | MYRF, PLP1, CNP | 0.647 |
| SN-M3 | 2.52E-26 | MOG, MYRF, CNP, MAG | SN-M4 | 4.61E-17 | MOG, CNP, MAG | 0.556 |

* Enrichment p-values were calculated by the Logistic regression method and adjusted using the BH procedure.

**Table S23. Statistics of ACGs by down-sampling male samples**

| Region | Mean APCGs (SD) | Mean ANCGs (SD) | *p*-value (APCGs) | *p*-value (ANCGs) |
| --- | --- | --- | --- | --- |
| AMY | 266.90 (469.94) | 297.91 (506.74) | 0.103 | 0.096 |
| ACC | 89.86 (298.07) | 89.87 (323.62) | 0.202 | 0.22 |
| CD | 593.54 (639.94) | 844.64 (898.10) | 0.097 | 0.083 |
| CBH | 81.25 (220.82) | 76.37 (199.80) | 0.026 | 0.031 |
| CB | 319.04 (432.33) | 245.92 (362.58) | 0.06 | 0.064 |
| CT | 566.26 (728.28) | 748.14 (912.19) | 0.181 | 0.177 |
| FC | 95.78 (306.53) | 96.53 (315.60) | 0.031 | 0.024 |
| HIPP | 780.60 (873.84) | 936.55 (999.92) | 0.046 | 0.031 |
| HTH | 620.80 (713.04) | 700.57 (812.58) | 0.022 | 0.048 |
| NAc | 103.41 (314.67) | 99.75 (314.75) | 0.175 | 0.165 |
| PT | 32.73 (183.07) | 31.34 (203.22) | 0.066 | 0.069 |
| SC | 27.28 (60.08) | 228.42 (415.31) | 0.159 | 0.158 |
| SN | 1.363 (12.23) | 0.98 (16.21) | 1 | 1 |

APCGs: Age positive correlated genes; SD, standard deviation

ANCGs: Age negative correlated genes; SD, standard deviation

**Table S24. Statistics of DEGs in subsets of male samples in aged versus young individuals**

| Region | Mean Up (SD) | Mean Dn (SD) | p.count+ | p.count- |
| --- | --- | --- | --- | --- |
| AMY | 245.78 (480.50) | 305.32 (560.48) | 0.165 | 0.146 |
| ACC | 85.57 (297.25) | 107.12 (369.78) | 0.457 | 0.427 |
| CD | 818.50 (769.97) | 1050.41 (974.05) | 0.152 | 0.173 |
| CBH | 54.24 (182.58) | 49.29 (166.50) | 0.074 | 0.078 |
| CB | 291.72 (508.69) | 238.49 (465.69) | 0.101 | 0.111 |
| CT | 501.48 (715.56) | 650.49 (893.26) | 0.18 | 0.168 |
| FC | 53.25 (168.21) | 44.96 (133.72) | 0.426 | 0.454 |
| HIPP | 417.52 (661.62) | 516.01 (788.71) | 0.089 | 0.073 |
| HTH | 819.29 (794.85) | 900.11 (895.35) | 0.014 | 0.023 |
| NAc | 99.88 (340.14) | 110 (374.27) | 1 | 1 |
| PT | 10.93 (109.71) | 10.33 (121.79) | 1 | 1 |
| SC | 8.91 (27.08) | 101.66 (269.95) | 1 | 1 |
| SN | 0.008 (0.10) | 0.065 (0.31) | 1 | 1 |

Column annotation

Up: Up-regulated genes in old age comparing to young adult

Dn: Down-regulated genes in old age comparing to young adult

**Table S25. Number DEGs in middle aged versus young individuals**

| **Region** | **Male** | | **Female** | |
| --- | --- | --- | --- | --- |
|  | **# Up DEGs** | **# Down DEGs** | **# Up DEGs** | **# Down DEGs** |
| AMY | 6 | 5 | 0 | 0 |
| ACC | 0 | 0 | 0 | 0 |
| CD | 598 | 373 | 55 | 20 |
| CBH | 0 | 0 | 1196 | 1141 |
| CB | 0 | 3 | 0 | 0 |
| CT | 0 | 0 | 0 | 0 |
| FC | 0 | 0 | 0 | 0 |
| HIPP | 0 | 0 | 0 | 0 |
| HTH | 6 | 4 | 0 | 0 |
| NAc | 0 | 0 | 0 | 0 |
| PT | 0 | 0 | 0 | 0 |
| SC | 0 | 0 | 0 | 0 |
| SN | 0 | 0 | 0 | 0 |

**Table S26. Number DEGs in aged versus middle aged subjects**

| **Region** | **Male** | | **Female** | |
| --- | --- | --- | --- | --- |
|  | **# Up DEGs** | **# Down DEGs** | **# Up DEGs** | **# Down DEGs** |
| AMY | 0 | 0 | 0 | 0 |
| ACC | 0 | 0 | 0 | 0 |
| CD | 28 | 8 | 0 | 0 |
| CBH | 17 | 5 | 0 | 0 |
| CB | 0 | 0 | 0 | 0 |
| CT | 1 | 0 | 2 | 1 |
| FC | 0 | 0 | 0 | 0 |
| HIPP | 2584 | 3101 | 0 | 0 |
| HTH | 9 | 5 | 0 | 0 |
| NAc | 683 | 474 | 0 | 0 |
| PT | 0 | 0 | 0 | 0 |
| SC | 0 | 0 | 0 | 0 |
| SN | 0 | 0 | 0 | 0 |
